# Supplementary material for: Optical Monitoring of the Biodegradation of Porous and Solid Silicon Nanoparticles
Source: Nanomaterials (Basel). 2021 Aug 25;11(9):2167. doi: 10.3390/nano11092167 (PMC8466475; doi:10.3390/nano11092167)
Supplement: Supplementary file 1 [file nanomaterials-11-02167-s001.zip › nanomaterials-1341677-SI.pdf]

nanomaterials

Supplementary Materials

Optical Monitoring of the Biodegradation of Porous and Solid Silicon Nanoparticles

Maxim B. Gongalsky [1,*], Nikolay V. Pervushin [2], Daria E. Maksutova [1], Uliana A. Tsurikova [1], Pavel P. Putintsev [1], Oleg D. Gyuppenen [1], Yana V. Evstratova [3], Olga A. Shalygina [1], Gelina S. Kopeina [2], Andrey A. Kudryavtsev [3], Boris Zhivotovsky [2,4] and Liubov A. Osminkina [1,5,*]

[1] Faculty of Physics, Lomonosov Moscow State University, 119991 Moscow, Russia; medar908@gmail.com (D.E.M.); natashina78@yandex.ru (U.A.T.); putintcev.pp19@physics.msu.ru (P.P.P.); guppenen19@mail.ru (O.D.G.); olga@ofme.phys.msu.ru (O.A.S.)
[2] Faculty of Medicine, Lomonosov Moscow State University, 119991 Moscow, Russia; rhododendron.nick@mail.ru (N.V.P.); lirroster@gmail.com (G.S.K.); boris.zhivotovsky@ki.se (B.Z.)
[3] Institute of Theoretical and Experimental Biophysics, Russian Academy of Sciences, 142290 Pushchino, Russia; yannaevstratova@gmail.com (Y.V.E.); centavr42@mail.ru (A.A.K.)
[4] Institute of Environmental Medicine, Karolinska Institutet, Box 210, 17177 Stockholm, Sweden
[5] Institute for Biological Instrumentation, Russian Academy of Sciences, 142290 Pushchino, Russia
* Correspondence: mgongalsky@gmail.com (M.B.G.), osminkina@physics.msu.ru (L.A.O.)

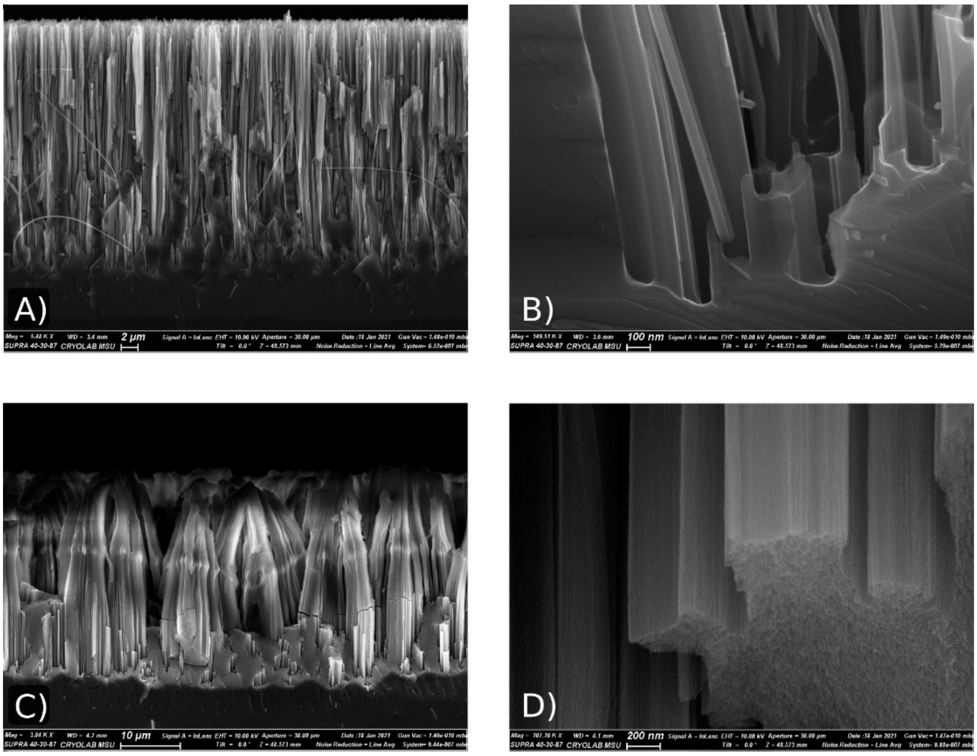

Figure S1. Electron microscopy images of layers of solid (**A**,**B**) and porous (**C**,**D**) silicon nanowires.

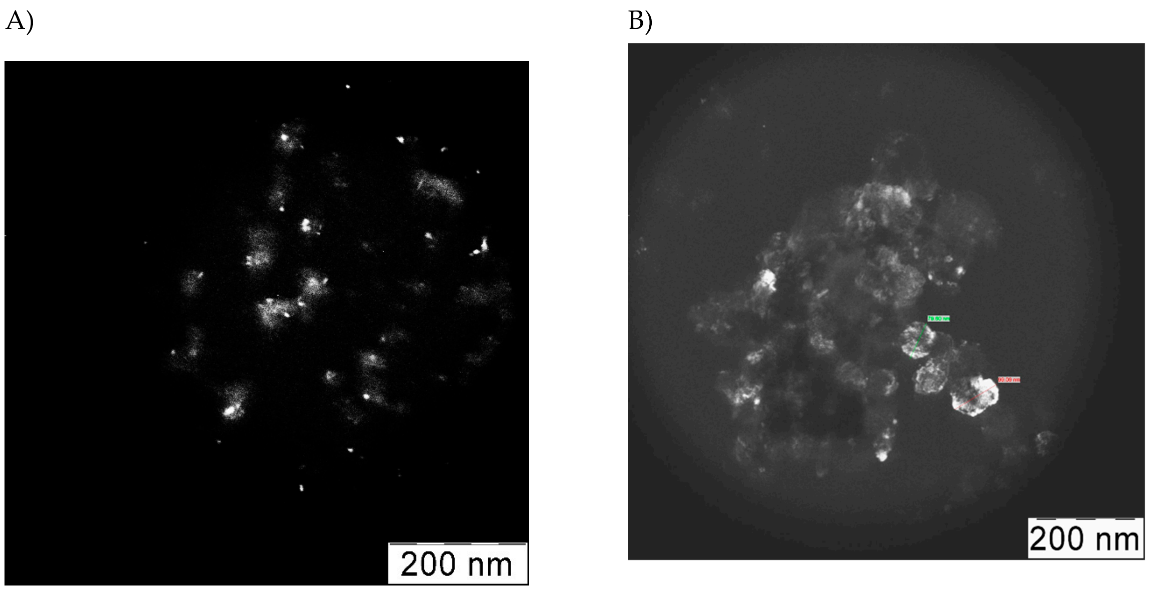

Figure S2. Dark field images of **A**) por-SiNPs and **B**) sol-SiNPs. Bright spots correspond to single nanocrystals. This image was used for calculation of nc-Si distributions.

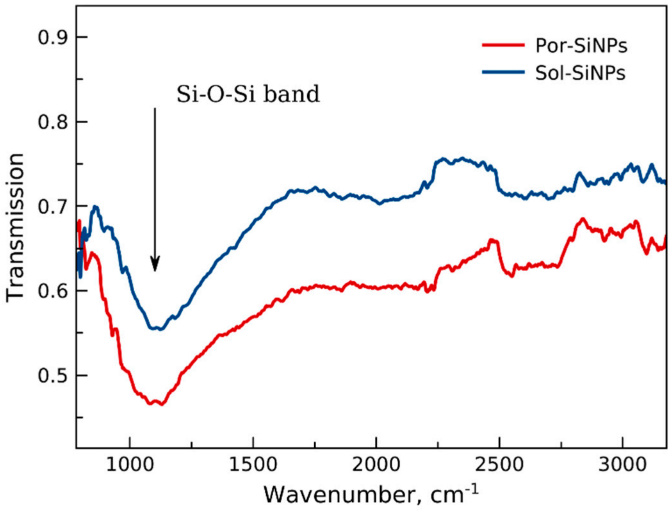

Figure S3. FTIR spectra of sol-SiNPs (blue) and por-SiNPs (brown) powders. Broad band at 1100 cm^{-1} corresponds to Si-O-Si oscillations.

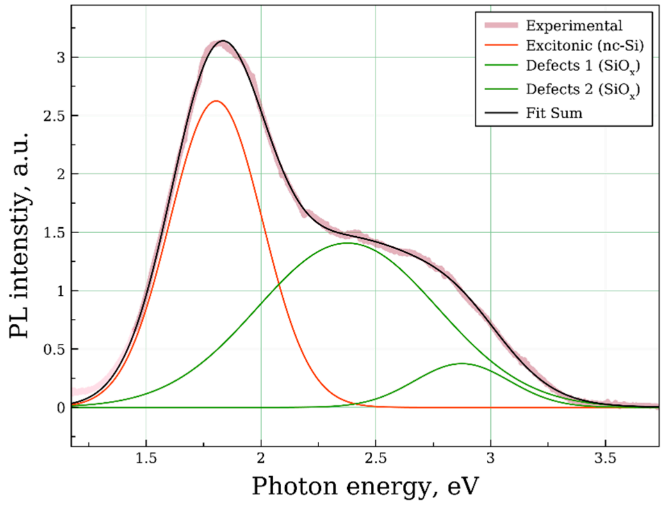

Figure S4. Example of PL spectrum deconvolution (por-SiNPs after 24 h) by low energy excitonic band at 1.8 eV (red), and two high energy defect bands in SiO_x shell.

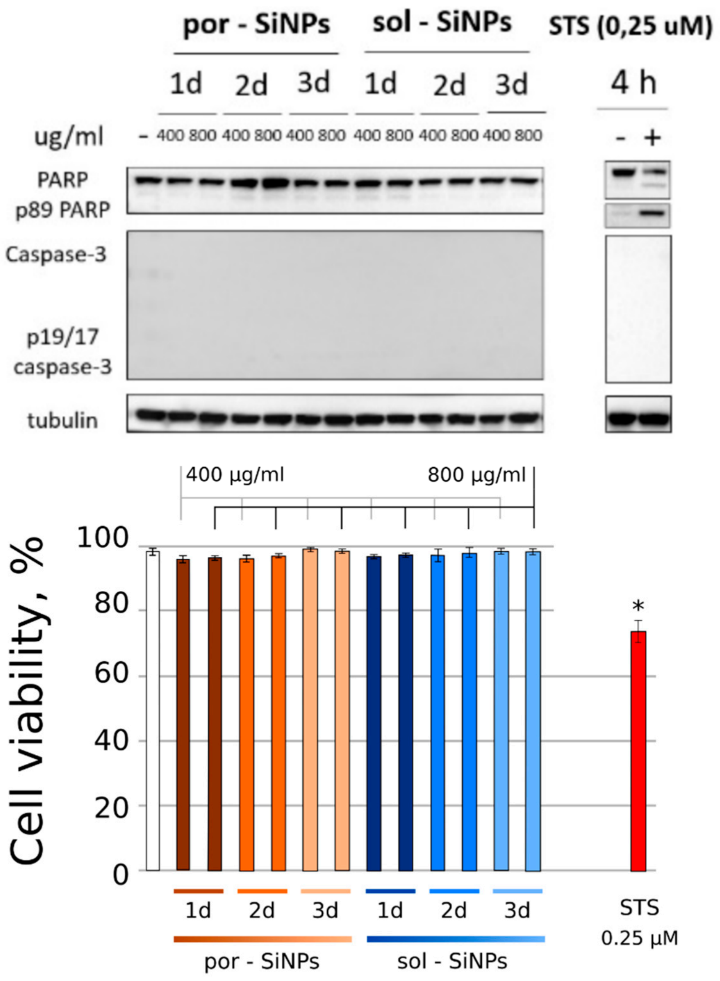

Figure S5. Top - Western Blot analysis of total cellular lysates from MCF-7 cells upon treatment with two types of nanoparticles (por-SiNPs and sol-SiNPs) and positive controls for apoptosis induction – STS. Tubulin was used as a loading control. Designations: PARP – full form and p89 fragment of PARP; Tubulin – alpha-tubulin; p19/17 caspase-3 – p19/17 fragments of caspase-3; STS – staurosporine in concentration 0,6 µM; h – hours, d - days. Bottom – The histogram of flow cytometry (FC) analysis data for MCF-7 cells treated with these nanoparticles at different concentrations and STS

(0,6 μM). All experiments were performed at least three times. Results are presented as mean +/- standard deviation (SD). *p < 0.05: significant difference compared to control cells (Mann-Whitney U test).

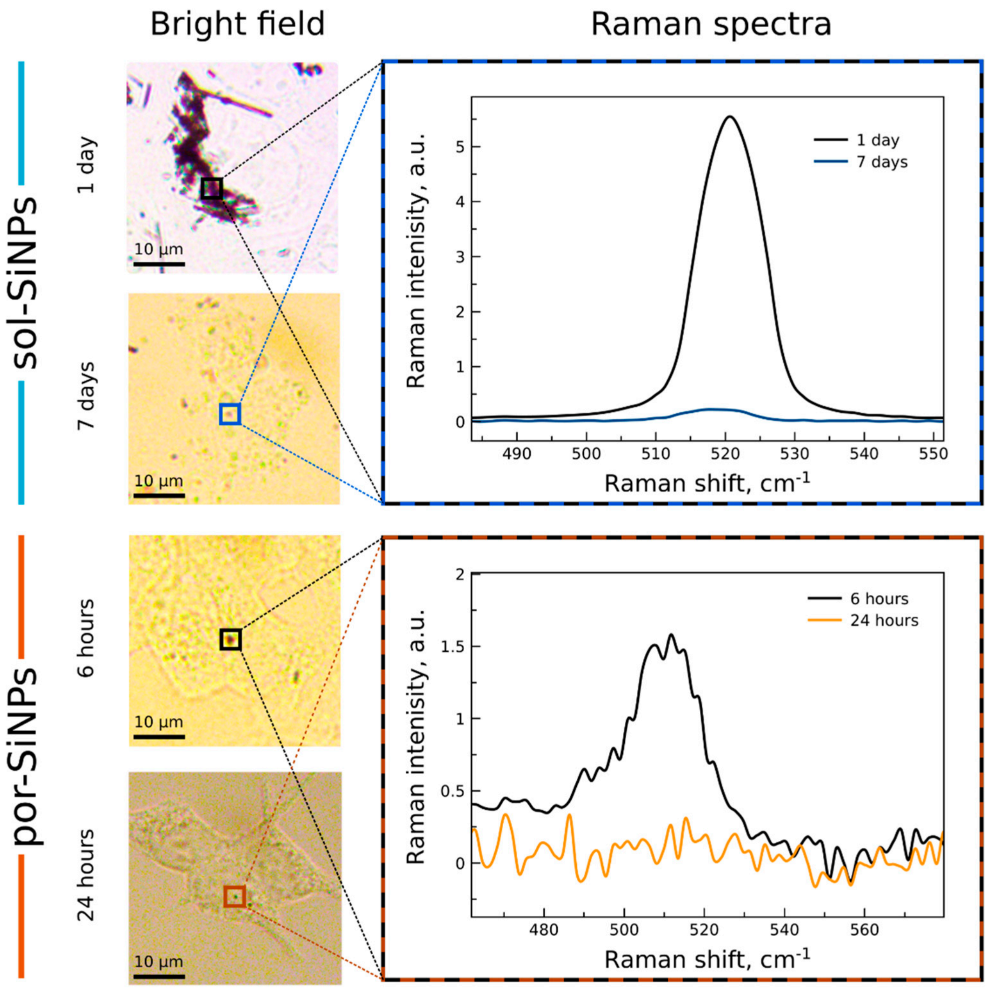

Figure S6. Widefield imaging and Raman spectra of SiNPs inside human cells (HEK293T). Left side: widefield images of sol-SiNPs after 1 and 7 days of incubation and por-SiNPs after 6 and 24 hours of incubation. Right side: corresponding Raman spectra of squared SiNPs.

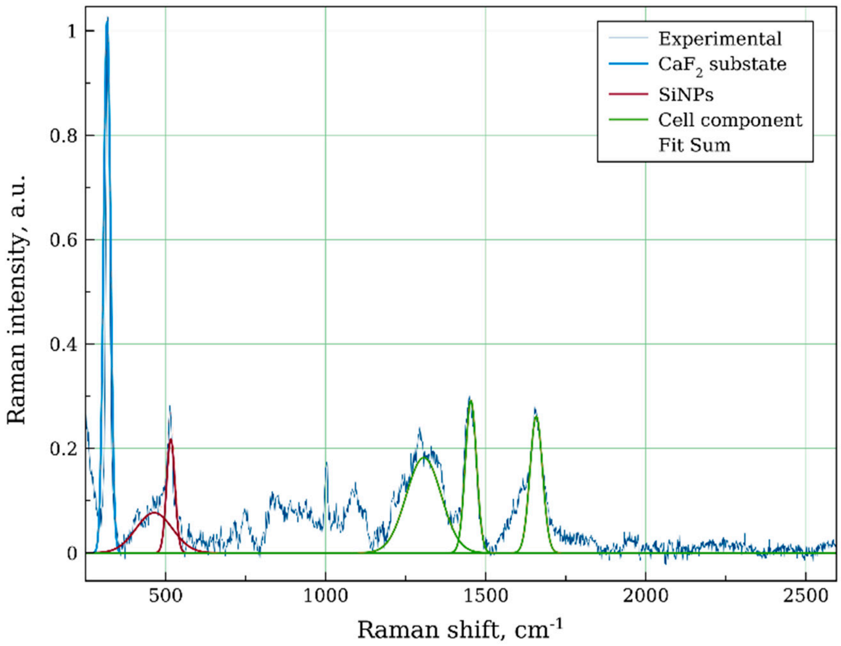

Figure S7. Example of Raman spectrum decomposition (por-SiNPs in HEK293T after 6 hours of incubation). Blue – CaF_2 substrate, brown – SiNPs (amorphous and nc-Si bands), green – cell components (proteins and lipids).

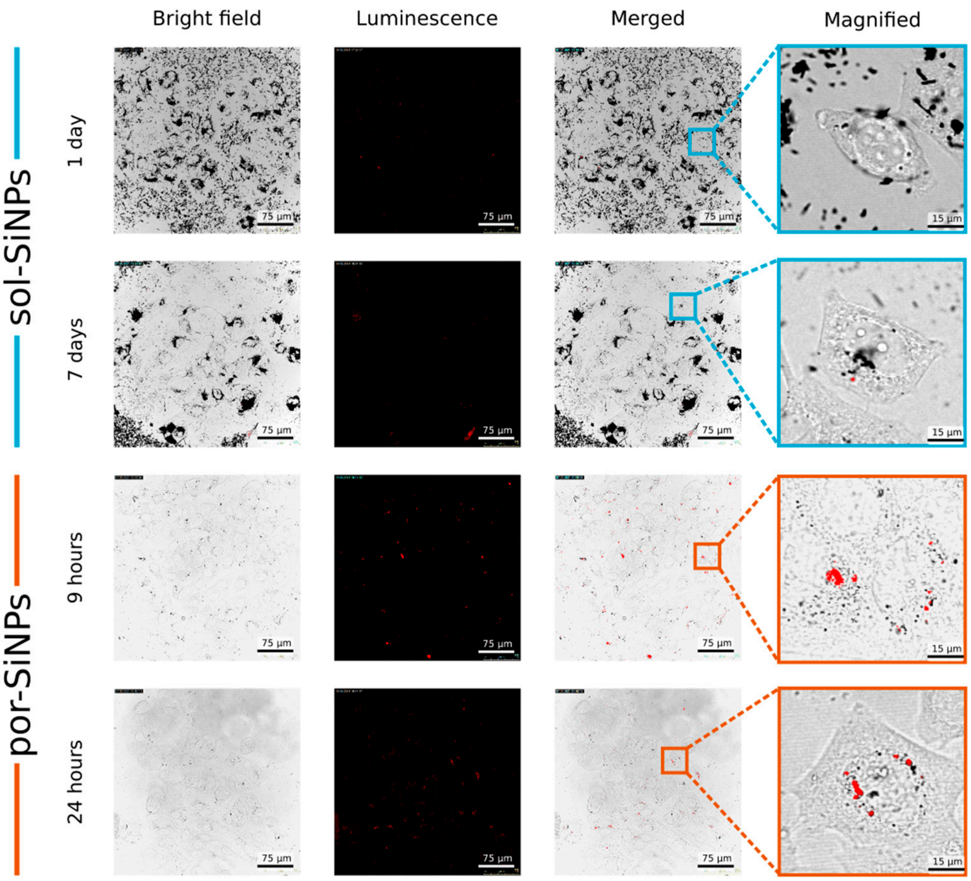

Figure S8. Widefield and luminescent confocal bioimaging of SiNPs inside human cells (MCF7). Columns (from left to right): widefield view of cells; luminescence; merged images; magnifications of merged images. Rows: sol-SiNPs after 1 and 7 days of incubation, and por-SiNPs after 6 and 24 hours of incubation.

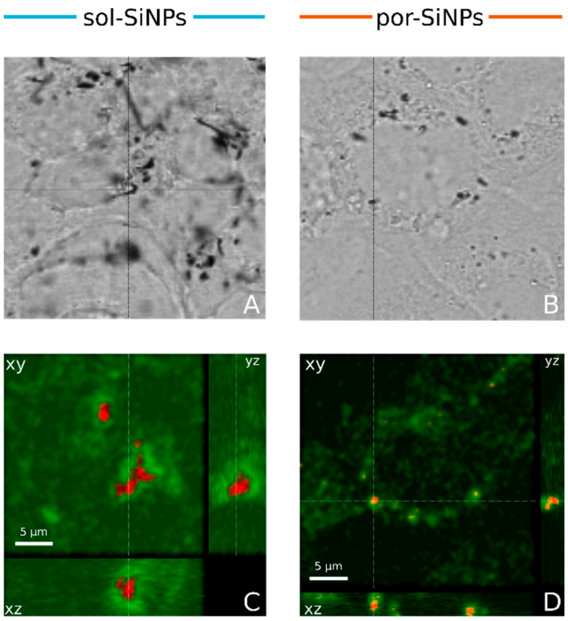

Figure S9. Demonstration of SiNPs internalization in cells (example). Widefield images of sol-SiNPs in HEK293T after 1 day of incubation (**A**), por-SiNPs in MCF7 after 9 hours of incubation (**B**). Confocal images of internalized sol-SiNPs (**C**) and por-SiNPs (**D**) in the same cells. Red spots correspond to SiNPs, green – to autofluorescence of cells. All three cross-sections in 3D space are shown in order to show that SiNPs are actually inside cells.

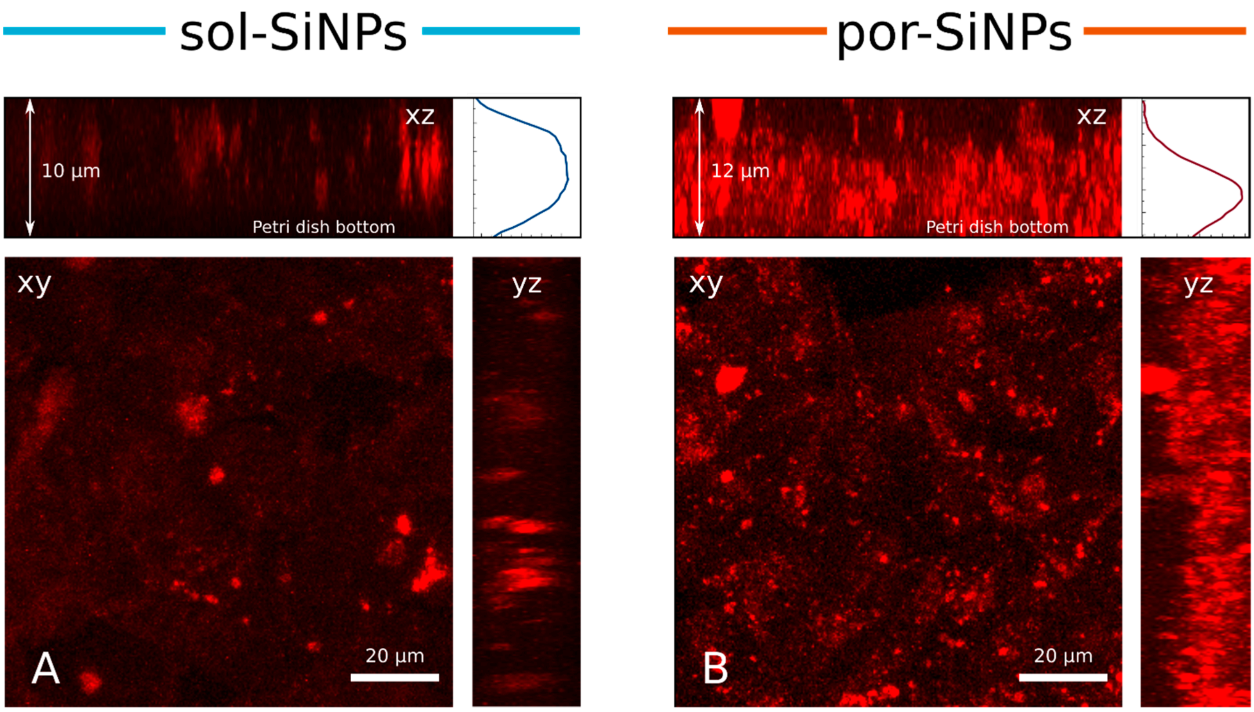

Figure S10. Second demonstration of SiNPs internalization inside cells. Confocal images of sol-SiNPs (**A**) and por-SiNPs (**B**) in HEK293T cells after 1 day and 9 hours of incubation, respectively. Three "maximum" projections are shown for each sample. Top panels, i.e. xz-projections are supplemented with average fluorescence intensity profile plots. Those profiles demonstrate preferable localization of SiNPs in the center of z-stack, which corresponds to average center of the cells and thus excludes the possibility that the majority of cells are deposited on the bottom of the Petri dishes or lie over the cells.
